# Prevalence of obesity and overweight among Chinese children with attention deficit hyperactivity disorder: a survey in Zhejiang Province, China

**DOI:** 10.1186/1471-244X-13-133

**Published:** 2013-05-10

**Authors:** Rongwang Yang, Shujiong Mao, Suhan Zhang, Rong Li, Zhengyan Zhao

**Affiliations:** 1 Department of Child Psychology, The Children’s Hospital, Zhejiang University School of Medicine, Hangzhou, Zhejiang Province, China; 2 Department of Pediatrics, The First People’s Hospital of Hangzhou, Hangzhou, Zhejiang Province, China; 3 Department of Child Health Care, The Children’s Hospital, Zhejiang University School of Medicine, Hangzhou, Zhejiang Province, China

**Keywords:** Attention deficit hyperactivity disorder, Obesity, Comorbidity, Children, Prevalence

## Abstract

**Background:**

Attention Deficit Hyperactivity Disorder (ADHD) is often comorbid with psychiatric and developmental disorders. This study aimed to investigate the prevalence of obesity and overweight among Chinese children with ADHD, and to explore which subtypes of the disorder may specifically be associated with obesity/overweight.

**Methods:**

Children meeting the DSM-IV criteria for ADHD were enrolled in the study. Weight, weight z-score, height, height z-score, BMI, and BMI z-score were used to evaluate growth status. Obesity and overweight were determined using the National Growth Reference for Chinese Children and Adolescents. Relations between the prevalence of obesity/overweight and different ADHD subtypes and pubertal development were analyzed.

**Results:**

A total of 158 children with ADHD (mean age: 9.2 years) were recruited for the study. The prevalences of obesity, overweight, and combined obesity/overweight were 12.0%, 17.1%, and 29.1%, respectively, which were significantly higher than in the general Chinese population (2.1%, 4.5%, and 6.6%, respectively). Multivariable analysis showed that the children with the combined subtype of ADHD and the onset of puberty were at a higher risk of becoming obese or overweight.

**Conclusions:**

The prevalence of obesity in Chinese children with ADHD is higher than that of the general population. Children with the ADHD combined subtype who were at the onset of puberty were more likely to be overweight or obese.

## Background

Attention deficit hyperactivity disorder (ADHD) is recognized as one of the most common childhood psychiatric disorders, and is characterized by age-inappropriate inattention, hyperactivity-impulsivity, or both [1,2].The condition is estimated to affect 5–10% of school-aged children worldwide, depending on different geographic areas, ethnic background, culture, and diagnostic criteria [3,4]. ADHD is often comorbid with psychiatric and developmental disorders such as anxiety disorders, oppositional defiant disorder, conduct disorder, and learning disorders [5-9], and is associated with impairment of academic and social functions [10,11].

In recent years, ADHD was found to be linked with another increasing worldwide childhood disorder: obesity [12-15]. The prevalence of ADHD in obese subjects is higher than that of the normal population. In 2002, Altfas [16] showed that 27.4% of obese adults were diagnosed with ADHD, which was higher than the prevalence in general adult subjects. In children and adolescents, similar results were obtained [14,15]. In addition, children and adolescents with ADHD had a higher body mass index (BMI) than expected for the age- and gender-specific group. Holtkamp et al. [17] found that the mean BMI standard deviation scores of children diagnosed with ADHD were significantly higher than the reference values of the normal German population. In 2007, Lam and Yang [18] reported that subjects with high scores of ADHD tendency (but not diagnosed with ADHD) had an increased risk for obesity of 1.4 times that of subjects with low ADHD tendency in the Chinese adolescent population. In a large sample survey in the United States of America, Waring and Lapane [19] reported that children with ADHD were much heavier than those without ADHD.

Although many studies suggest a potential comorbidity between obesity and ADHD, other results have shown that the rates of overweight and obesity among children with ADHD were not higher than the rates in the general population [20,21]. However, most of the studies were performed in specific subgroups [16,17,22-24], such as American adults and children, German adults and children, Turkish adolescents, and so on. Research has shown that individuals from diverse ethnic backgrounds engage in lifestyle practices that may influence the prevalence of both conditions [3,12], so it is important to investigate the possible comorbidity in populations with different ethnic and cultural backgrounds. To our knowledge, the prevalence of obesity and overweight in Chinese children with a diagnosis of ADHD has not been explored. One study conducted in China in 2007 by Lam and Yang [18] showed a close relationship between ADHD tendency and obesity. However, it was evaluated in a general Chinese population of adolescents, not in an ADHD-diagnosed group.

Taking this research into consideration, the present study was performed in Zhejiang Province, China. We hypothesized that the prevalence of obesity was higher in ADHD patients, and that a specific subtype of ADHD was associated with obesity. Our aims were as follows: 1) to investigate the prevalence of obesity or overweight among Chinese children with ADHD, and to compare this prevalence with what is reported in the general Chinese population, and 2) to analyze the prevalence of obesity/overweight in children with different subtypes of ADHD to identify which dimension(s) of ADHD may specifically be associated with obesity/overweight.

## Methods

### Study design

The data for this study were collected from outpatients who were believed to be affected by ADHD on their first visit to our clinic between March 1, 2010 and August 31, 2010. Patients visited this clinic only for psychological problems. No children who simply displayed obesity or overweight visited the clinic. The hospital has a separate center for the diagnosis and treatment of children with obesity and overweight. Therefore, sampling bias was avoided in this study. Participants who were diagnosed with any subtype of ADHD [1] were included. Growth assessment of the children was performed after the study procedures were explained and oral consents were obtained from them and their parents. The study protocol was approved by the Institutional Review Board of the Children’s Hospital, Zhejiang University School of Medicine.

### Participants and diagnostic assessment

Subjects with ADHD were eligible to participate in the study at our clinic at the Children’s Hospital, Zhejiang University School of Medicine, which specializes in evaluating and treating children with behavioral, developmental, and psychiatric disorders. The children underwent a comprehensive assessment process by a trained multidisciplinary ADHD diagnostic team, including physical examination, EEG, ECG, Vanderbilt ADHD Diagnostic Parent and Teacher Rating Scales, Conners’ Parent and Teacher Rating Scales (Chinese version), SNAP-IV, and Wechsler Intelligence Scale for Chinese Children (WISC-R) [25]. The children, parents, and teachers were interviewed by one psychiatrist to verify the ADHD diagnosis.

Children included in this study met the following criteria: (a) diagnosed with ADHD according to the Diagnostic and Statistical Manual of Mental Disorders, Fourth Edition (DSM-IV) [1] criteria, under the following three subtypes: ADHD inattentive type, ADHD hyperactive-impulsive type, and ADHD combined type; (b) between the ages of 6 and 16 years; (c) having a full scale IQ above 80 based on the WISC-R [25]; (d) having no history of treatment for ADHD; and (e) being Han Chinese.

Individuals were excluded if they had: (a) a psychiatric diagnosis with significant symptoms (psychosis, autism, depression, anxiety, mood disorder) and mental retardation; (b) sensorimotor handicaps (paralysis, deafness, blindness); (c) taken drugs such as methylphenidate in any form, clonidine, or atomoxetine for controlling ADHD symptoms; or (d) serious somatic disorders, such as cardiovascular diseases, hyperthyroidism, serious gastrointestinal stenosis, dysphagia, exogenous steroid hormones, and sexual development disorders.

### Growth measurement and assessment

Growth data were taken during the first recruitment visit by one assistant. Body weight was measured to the nearest 0.10 kg while the subjects were wearing lightweight clothing, and height was measured to the nearest 0.10 cm without shoes and hats.

The indicators of weight percentile, weight z-score, height percentiles, height z-score, BMI percentiles, and BMI z-score were used to determine the individuals’ growth, which was evaluated using the National Growth Reference for Chinese Children and Adolescents [26]. Individuals’ weight percentile was determined according to the age- and gender-specific standardized growth chart. Weight z-score was defined as the difference between a subject’s absolute weight and the mean weight, for children of the same age and gender, divided by the standard deviation of the weight for that subgroup. BMI was calculated by dividing the subject’s weight (in kg) by the square of his or her height (in meters). Height percentile, height z-score, BMI percentile, and BMI z-score were defined by the same processes as weight percentile and weight z-score. Age in months was calculated from the date of birth to the date of the visit.

We referenced the growth chart for Chinese children and adolescents aged 0–18 years as the standardized tool to define normal weight, overweight, and obesity [26,27]. This growth chart was developed based on the nationally representative survey of the Physical Fitness and Health Surveillance of Chinese School Students in 2005 [26], and reflects the latest growth data of Chinese children aged 0–18 years in China. According to the manual, cutoff points were chosen based on the Working Group on Obesity in China (WGOC) for overweight and obesity, which were close to the 85^th^ and 95^th^ BMI percentiles.

### Pubertal development

To assess the stage of pubertal development, children older than 8 years were asked questions about pubertal development. These included questions about the presence or absence of pubic hair, axillary hair, facial hair, and nocturnal emission for males; and pubic hair, axillary hair, and menses for females; as well as the age of attainment of each stage. Based on these questions, estimates of the individuals’ pubertal developmental stages were defined as follows: absence of pubic hair and any other sign of puberty was the pre-pubertal stage; attainment of any sign of puberty was the pubertal stage.

### Data analysis

Data were entered into Excel software and analyzed in the SPSS (version 13.0) software package. Before a one-sample *t*-test was used to compare the z-scores of height, weight, and BMI to the mean value of 0, the normal distributions of the sample values were tested. Differing gender and pubertal developmental stages were both categorical data, and were analyzed by chi-square analysis or Fisher’s Exact Test, for any analysis with a count in a cell of fewer than 5. A P value of less than 0.05 was regarded as statistically significant for all statistical tests. Three categories of independent variables considered to be associated with the prevalence of obesity/overweight were examined: (1) gender, (2) pubertal developmental stage, and (3) subtypes of ADHD. We used univariate analysis to quantify the association between variables and obesity. Variables found to be statistically significant in univariate analysis were used to establish a multivariable logistic regression model with a forward stepwise method to determine the risk factors for obesity.

## Results

There were 162 children diagnosed with ADHD between March 1, 2010 and August 31, 2010. Four children were excluded from the study because either their consents were not obtained, or the information they provided was incomplete. The characteristics of the subjects are summarized in Table 1. The mean age was 9.2 years, with standard deviation of 2.0 (they ranged from 6–16.6 years), and most (94.3%, n = 149) were in primary school. Of the children with ADHD, 79.7% (n = 126) were from 7–10 years of age, 7.0% (n = 11) were younger than 7 years, and 13.3% (n = 21) were older than 11 years. The sample consisted mainly of children with ADHD inattentive subtype (50.6%, n = 80). There were more males (77.8%, n = 123) than females. The ratio was 3.5:1, similar to other epidemiologic reports [3].

**Table 1:** General information and frequency distributions of children with ADHD (n = 158)

***Variables***	***Frequency***	***Percentage (%)***
**Demographics**		
*Gender*		
*Male*	123	77.8
*Female*	35	22.2
*Age groups*		
*6 years*	11	7.0
*7-10 years*	126	79.7
*Older than 11 years*	21	13.3
Mean age (SD)	9.2 (2.0)	
*Developmental stage*		
*Pre-pubertal*	141	89.2
*Pubertal*	17	10.8
**ADHD subtypes**		
*Inattentive*	80	50.6
*Hyperactive/Impulsive*	33	20.9
*Combined*	45	28.5

ADHD: Attention deficit hyperactivity disorder; SD: Standard deviation.

The prevalences of obesity, overweight, and combined obesity/overweight were 12.0% (19/158), 17.1% (27/158), and 29.1% (46/158), respectively, in this group of children with ADHD. The prevalence depended on the pubertal developmental stage and ADHD subtype (Table 2).

**Table 2:** Prevalence of overweight and obesity in the children of the ADHD group in relation to different categories

	**Overweight (%)**	**Obesity (%)**	**Overweight + Obesity (%)**
**Gender**			
*Male*	20 (16.3%)	15 (12.2%)	28.5
*Female*	7 (20.0%)	4 (11.4%)	31.4
**Developmental stage**			
*Pre-pubertal*	23 (16.3%)	13 (9.2%)	24.1
*Pubertal*	4 (23.5%)	6 (35.3%)	58.8
**ADHD subtypes**			
*Inattentive*	10 (12.5%)	10 (12.5%)	25.0
*Hyperactive/Impulsive*	3 (9.1%)	4 (12.1%)	21.2
*Combined*	14 (31.1%)	5 (11.1%)	42.2
**Total**	27 (17.1%)	19 (12.0%)	29.1

ADHD: Attention deficit hyperactivity disorder.

Anthropometric data of the study samples, including the distribution of height and BMI in percentile, are presented in Table 3. The mean z-scores for the children’s height, weight, and BMI were -0.12 ± 1.01, 0.05 ± 1.36, and 0.20 ± 1.71, respectively. The mean BMI z-score (0.20 ± 1.71) of the children with ADHD was greater than 0. However, the difference did not reach statistical significance (P = 0.147). The proportion of children with a BMI below the 5^th^ percentile was 8.23% (13/158).

**Table 3:** Anthropometric data of ADHD children

**Category**	***ADHD group***
	**Frequency**
***BMI (percentile)***	
*--5*^*th*^	13
*6*^*th*^*--50*^*th*^	49
*51*^*st*^*—95*^*th*^	77
*95*^*th*^*--*	19
***Height z-score (*Mean ± SD*)***	-0.12 ± 1.01
***Weight z-score (*Mean ± SD*)***	0.05 ± 1.36
***BMI z-score (*Mean ± SD*)***	0.20 ± 1.71

ADHD: Attention deficit hyperactivity disorder; BMI: Body mass index; SD: Standard deviation.

Univariate analysis showed that three potential risk factors were associated with obesity/overweight. Children in the pubertal stage were more likely to be overweight/obese (OR = 3.162, P = 0.027). Children with ADHD combined subtype had a greater possibility of being overweight/obese (OR = 2.192, P = 0.048). Gender was not a risk factor for obesity/overweight.

Multivariate analysis (Table 4) demonstrated that children in puberty who had ADHD had a four-fold increase in the odds ratio of obesity/overweight than those in the pre-pubertal stage (95% CI: 1.337-12.191). Children with ADHD combined subtype were 2.8 times more likely to be obese/overweight than those with either of the other two ADHD subtypes (95% CI: 1.225–6.434).

**Table 4:** Risk factors for children with ADHD and obesity/overweight (multivariate logistic regression analysis) (n = 158)

**Variable**	**Odds ratio**	**95% Confidence interval**	**P-value**
Developmental stage (pre-pubertal vs pubertal)	4.098	1.337-12.191	0.011
ADHD subtype			
ADHD-C	2.808	1.225-6.434	0.015

ADHD: Attention deficit hyperactivity disorder; ADHD-C: ADHD combined type.

## Discussion

In the present study, we found that the prevalence of obesity/overweight in Chinese children in Zhejiang Province with ADHD is 29.1%, which is higher than the figure of 6.6% found in the national survey of the normal population in 2002 [28]. When we compared our results with the survey conducted in Zhejiang Province in 2005 (published in 2006), the finding was similar [29]. Table 5 shows the recent surveys on the prevalence of pediatric obesity throughout China [28-38]. The present data reveal that Chinese children with ADHD have a higher prevalence of obesity/overweight. We re-evaluated the children’s growth data using the World Health Organization criteria. The rate of overweight was 15.8% (25/158), and that of obesity was 11.4% (18/158). This prevalence was much higher than that reported in other Provinces of China (Table 5), as well as that in Zhejiang Province reported by Yang [29]. In addition, the absolute mean BMI z-score of children with ADHD is larger than 0, which confirms that the children with ADHD are more likely to be associated with obesity/overweight. Our finding is in line with previous reports by others [18,22,23] and strongly suggests that obesity is another comorbidity of ADHD in Chinese children.

**Table 5:** Prevalence of obesity and overweight in normal Chinese children

**Authors**	**Published year**	**Sample resource**	**Sample size**	**Age group (y)**	**Obesity (%)**	**Overweight (%)**	**Reference criteria**
Wu [28]	2006	National	44880	7-17	2.1	4.5	WGOC
Shan et al. [30]	2010	Beijing (Northern China)	19517	6-18	9.8	11.9	WGOC
Ying-Xiu et al. [31]	2011	Shandong Province (Eastern China)	7577	7-18	11.5	14.6	WGOC
Tang et al. [32]	2011	Guangxi Province (Southern China)	117230	7-18	4.2	12.7	WGOC
Chen et al. [33]	2010	Heilongjiang Province (Northeastern China)	860	7-17	9.4	11.7	WGOC
Wang et al. [34]	2008	Sichuan Province (Southwestern China)	6345	6-18	9.2	9.6	WHO
Jiang [35]	2005	Hubei Province (Central China)	6098	7-12	7.46	--	WHO
Sun et al. [36]	2004	Tianjin (Northern China)	9908	2-16	7.4	--	WHO
Yang [29]	2006	Zhejiang Province (Eastern China)	3536	6-13	4.3	6.3	WHO
Luo et al. [37]	2004	Shanghai (Eastern China)	65006	6-18	3.3	12.95	IOTF
Li et al. [38]	2010	Shanxi Province (Northwestern China)	1792	11-17	3.6	12.7	IOTF

WGOC: Working group on obesity in China; WHO: World health organization; IOTF: International obesity task force.

Here, we want to emphasize several strengths of this study. First, our study was conducted in children who were diagnosed with ADHD for the first time and who were medication-naive, which minimized the potential influence of drugs on growth status [39,40]. Second, BMI z-score was employed as an indicator, which was a practical, convenient, and widely accepted screening tool and could be used to compare different age groups, avoiding mathematical distortion [41]. Third, we used the latest Chinese reference for children’s and adolescents’ growth to assess growth status, which could define the growth level more accurately. All of these points strengthen the results with more persuasiveness [26].

Several hypotheses [12,42] have tried to explain the potential mechanism of ADHD and obesity, such as an imbalance in the dopaminergic reward system [43] and excessive daytime sleepiness [44]. For the imbalance of the dopaminergic reward system, an insufficient dopamine-related natural reward leads to the use of “unnatural” immediate rewards, like inappropriate eating [45]. Changes in dopamine receptor (DR) D2 [46] and DRD4 [47,48] are found to be associated with this imbalance in the dopaminergic reward system in obese patients [49,50] and ADHD patients [49,51,52]. Therefore, obesity and ADHD may result from this common pathway of dysfunctions in the dopaminergic system. For sleep problems, alertness alterations, such as sleep apnea and daytime sleepiness, have been found in ADHD as well as in obesity [53-55]. It was hypothesized that excessive daytime sleepiness might begin to explain the association between ADHD and obesity [44], which had been confirmed in one study [56]. However, this hypothesis has a long way to go to explain this mechanism. However, if a subtype of ADHD (inattention, hyperactivity/impulsivity, or combined type) is found to be associated with obesity, the connection could offer an understanding of the underlying mechanism. The specific relationship is still unclear, though, as there are only a few reports on the subject [57,58]. In our survey, children with ADHD combined type were 2.8 times as likely to be obese/overweight as those with the other two ADHD subtypes. It is difficult to explain our results using the existing theory. Previous studies suggested that ADHD impulsivity might foster abnormal eating behaviors, which may contribute to maintaining a sufficient energy balance and the development of obesity [58]. In addition, the inattention symptoms are closely related to poor executive function, resulting in irregular eating patterns and obesity. It is proposed that hyperactivity-impulsivity and inattentive behaviors have cooperation effects on the subjects that would develop into obesity, yet individually the aspects contributed little to obesity.

Interestingly, this study found that pubertal development was associated with a higher prevalence of obesity/overweight in children with ADHD. When the sample of the ADHD group was restricted, children in puberty were associated with a four-fold increase in the odds ratio of obesity/overweight compared with those in the pre-pubertal stage. van Egmond-Frohlich and colleagues [59] found that ADHD symptoms (assessed by the Strengths and Difficulties Questionnaire with the hyperactivity/inattention subscale) were related to overweight in adolescent girls. Our results confirmed the association in a clinic-based sample of pubertal children, but were not confined to pubertal girls because the present survey did not allow us to calculate that specific association. It was speculated that other factors, in addition to impulsive and inattentive symptoms, or perhaps hormones affecting children with ADHD, play important roles in producing obesity during puberty.

Another interesting finding was that the proportion of children with BMI below the 5^th^ percentile was 8.23% (13/158), suggesting that the prevalence of underweight was also higher in the ADHD group. Most of them were males (12/13) who were diagnosed with ADHD inattentive type (8/13), followed by ADHD hyperactive-impulsive type (3/13), and ADHD combined type (2/13). This result was first reported in a retrospective survey with only three cases [60] that showed two of the three ADHD patients were underweight. Because our samples were medication-naive, the possibility that medications induced underweight by appetite suppression was excluded. We speculated that the hyperactivities resulted in excessive energy expenditure, which contributed to underweight.

The potential limitations of this study should be taken into consideration. First, the cross-sectional design precludes determining any causal inferences in the relationship between ADHD and obesity. Second, no control group was set to compare with the study group. However, in addition to comparing our results with the national survey, other investigations in China were used for comparison, including one conducted in Zhejiang Province, which could produce relatively objective conclusions. It should also be taken into consideration that the prevalence of obesity is rising quickly, and the data may not be completely up to date. Third, demographic and family information, obesity family history, and other parental characteristics of the ADHD group were not collected, and our population may have different lifestyles, food sources, and general structure, which would affect the children’s nutritional status. Finally, pubertal stages were estimated based on self-report, and data on the variety of pubertal stages were not collected, which might produce bias and affect the results. Therefore, a scientific design using a multi-center and random sampling method with larger sample size could be used to better explore this comorbidity.

## Conclusions

Our findings demonstrated that the prevalence of obesity in children with ADHD was higher than in the general population, which strongly supports obesity as another comorbidity of ADHD in Chinese children. Children with ADHD of the combined subtype and in puberty had a higher risk for obesity/overweight. These findings extend the comorbidity of ADHD and obesity to Chinese populations, consistent with previous findings reported in other populations.

## Competing interests

The authors declare that they have no competing interests.

## Authors’ contributions

RY and ZZ were responsible for the conception and design of the study. RY and SM performed the data analysis. All authors participated in interpretation of the findings. RY and SZ drafted the manuscript. RL and ZZ revised and commented on the draft and all authors read and approved the final version of the paper.

## Pre-publication history

The pre-publication history for this paper can be accessed here:

http://www.biomedcentral.com/1471-244X/13/133/prepub
